# The Efficiency of Operating Microscope Compared with Unaided Visual Examination, Conventional and Digital Intraoral Radiography for Proximal Caries Detection

**DOI:** 10.1155/2009/986873

**Published:** 2009-01-29

**Authors:** Ilkay Peker, Meryem Toraman Alkurt, Oya Bala, Bulent Altunkaynak

**Affiliations:** ^1^Department of Oral Diagnosis and Radiology, Faculty of Dentistry, Gazi University, 06490 Ankara, Turkey; ^2^Department of Operative Dentistry, Faculty of Dentistry, Gazi University, 06490 Ankara, Turkey; ^3^Department of Statistics, Faculty of Arts and Sciences, Gazi University, 06490 Ankara, Turkey

## Abstract

*Objective*. The purpose of this study was to evaluate the efficiency of operating microscope compared with unaided visual examination, conventional and digital intraoral radiography for proximal caries detection. *Materials* *and Methods*. The study was based on 48 extracted human posterior permanent teeth. The teeth were examined with unaided visual examination, operating microscope, conventional bitewing and digital intraoral radiographs. Then, true caries depth was determined by histological examination. The extent of the carious lesions was assessed by three examiners independently. One way variance of analysis (ANOVA) and Scheffe test were performed for comparison of observers, and the diagnostic accuracies of all systems were assessed from the area under the ROC curve (*A*_*z*_). *Results*. Statistically significant difference was found between observers (*P* < .01). There was a statistically significant difference between operating microscope-film radiography, operating microscope-RVG, unaided visual examination-film radiography, and unaided visual examination-RVG according to pairwise comparison (*P* < .05). *Conclusion*. The efficiency of operating microscope was found statistically equal with unaided visual examination and lower than radiographic systems for proximal caries detection.

## 1. Introduction

A variety of test methods are discussed for the diagnosis of proximal tooth surfaces. Adjuncts such as bitewing radiography and fiber-optic transillumination provide an improvement to unaided vision. Unaided visual diagnosis had detected fewer than 50% of caries lesions on occlusal surfaces and even fewer on proximal surfaces [1].

It is not possible to detect only with unaided visual examination in interproximal caries lesions; radiographs help for proximal caries diagnosis and detection of their lesion depth [2, 3]. The combination of visual inspection and bitewing radiographic images is accepted as a standard procedure in proximal caries diagnosis [4]. However, proximal radiolucencies on bitewing radiographs are not always indicative of clinical cavitation. The deeper the radiolucency penetrates enamel and dentine, the higher the probability of cavitation [5].

Due to difficulties in proximal caries detection, different methodologies were investigated. Magnification is an accessible, commonly advocated aid to diagnosis [6]. Recently, the new methods of magnifying visual aids such as intraoral camera, magnification loops, and operating microscope are used for caries diagnosis, restorative treatment decisions, root resection, and retrograde canal preparation [7, 8]. Previous studies [9, 10] had investigated the efficiency of operating microscope for occlusal caries diagnosis, but there is insufficient publication [5, 11] about usage of this device for proximal caries detection in dental literature.

The purpose of this study was to evaluate the efficiency of operating microscope compared with unaided visual examination, conventional and digital intraoral radiography for proximal caries detection by means of receiver operating characteristic (ROC) curve analysis.

## 2. Materials and Methods

The study was based on 48 extracted human posterior permanent teeth, 24 molars and 24 premolars stored in a 5% buffered formalin solution. No specimens exhibited any restoration on the proximal surfaces. Organic and inorganic debris were removed by an excavator and then the teeth were cleaned by pumice and water slurry. Three mouth models were prepared with the teeth to simulate the clinical condition. The models were fixed in a phantom head which was adjusted to a dental unit during the sessions of unaided visual examination and operating microscope assessment. The proximal surfaces coronal to the cementoenamel-junction of the teeth were assessed by two specialists of oral diagnosis and radiology and one specialist of restorative dentistry of at least 10 years of experience independently. To avoid observer fatigue, an interval of at least one week had separated each diagnostic session.

The models were examined under a dental unit light, by using a dental mirror (size 5) and the air water syringe of the dental unit without any magnification for unaided visual examination. The clinicians evaluated the extent of the carious lesions in the proximal surfaces of the teeth according to a 5-point rating scale (Table 1) [5].

Then the teeth were examined using an operating microscope 16x magnification (Moller-Wedel, Dento 300, Wedel, Germany) according to the same scale. The observers assessed the teeth adjusting the height of the operating stool at a 12 o'clock position. The position of operating microscope was not changed to eliminate the position errors during the examinations. Pictures captured on the computer monitor were recorded using a video recorder.

After unaided visual and operating microscope examinations were completed, the teeth were mounted in dental stone models 3 in a row (either 2 premolars and 1 molar or 1 premolar and 2 molars) with proximal surfaces in contact.

Conventional bitewing radiographs of the teeth were obtained using a specially designed holder to provide standardized bitewing projection geometry in the buccolingual direction, tangential to the proximal surfaces. The object to film distance was approximately 0.5 cm and the source-to-image receptor distance was 32 cm. Size 2 Insight (Eastman Kodak Company, Paris, France) films with an exposure time of 0.16 seconds and CCX intraoral unit (Trophy, Instrumentarium, Tuusula, Finland) with focal spot of size 0.8 mm, operating at 70 kVp and 8 mA, with 2.5 mm of aluminum-equivalent filtration were used. One centimeter of soft tissue equivalent material was used to simulate scatter radiation and beam attenuation from facial tissues. All film radiographs were developed in automatic film processor (Velopex, Extra-X, Medivance Instruments Ltd., London, UK, and NW107A) with freshly prepared solutions in the same day.

The CCD-based system to be evaluated was the Radiovisiography (RVG, 2000 Model, Trophy Radiologie, Paris, France). Digital images were obtained with 32 cm sensor to focal spot distance with an exposure time of 0.08 seconds under the same standardized conditions and were stored using the RVG image management software.

The film radiographs were assessed using a masked light box and a 2x magnification X-viewer (Luminosa, CSN Industrie, Cinisello Balsamo, Italy) by three clinicians independently in a quiet room with subdued ambient lighting. Images from the digital system were displayed on a 17-inch monitor in the same ambient lighting. Brightness and contrast features of the software were not changed. The observers indicated their decision separately for each interproximal side of the teeth by masking other side with the use of a black cartoon. They assessed the extent of the carious lesions according to a 5-point rating scale (Table 1) [12].

After all assessments were completed, the teeth were histologically prepared. The proximal surfaces were first colored with a solution of propylene glycol with added basic fucsin (0.5%) for 10 seconds and rinsed in tap water. Then, the teeth were hemisectioned perpendicularly to the proximal surfaces from their santral fossas by a diamond disc under water-cooling. Two sections were obtained, each section was examined under stereomicroscope (Olympus SZ 60, Tokyo, Japan) with a 10x magnification. Two observers not participating in the study both experienced in histological examination and being blinded to the radiographic appearance of the surfaces evaluated the sections by consensus according to a 5-point confidence scale (Table 1) [12].

Histological validation served as a “gold standard” for all tested methods. One way variance of analysis (ANOVA) and pairwise comparisons (Scheffe test) were performed for comparison of observers. The diagnostic accuracies of the four diagnostic systems were assessed from the area under the ROC curve (*A*_*z*_). Med-Calc (version 7.3) was used for ROC analysis. The rating scales were dichotomized as “presence” or “absence” of caries during the analysis. Score 0 in both radiographic and histological scales was detected as absence of caries and the others were detected as presence of caries. *A*_*z*_ values were calculated for each observer for each diagnostic method. The *A*_*z*_ values were analyzed by pairwise comparison of ROC curves. SPSS-version 13.0 for Windows was used for all calculations. The level of statistical significance was *α* = 0.05.

## 3. Results

The status of the 96 proximal surfaces of the teeth were assessed. Histological examination of the teeth confirmed that 61 (63.54%) of the proximal surfaces were caries free, whereas 35 (36.46%) of proximal surfaces determined caries lesions of different depths. The numbers of proximal surfaces for each score according to the histological examination are shown in Table 2.

Statistically significant difference was found between three observers at 99% confidence interval (*P* < .01) according to ANOVA. Scheffe test from pairwise comparisons was performed to determine which observers were different. No statistically significant difference was found between 1st and 2nd observers (*P* < .05) and there was statistically significant difference between both 1st and 3rd observers and 2nd and 3rd observers (*P* < .01) (Table 3).

**Figure 1:**
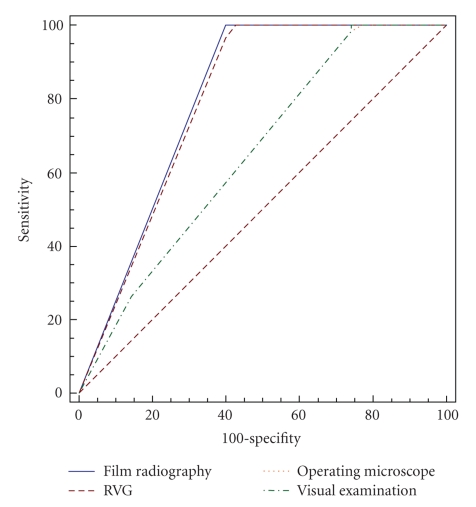
ROC curve for 1st observer.

**Figure 2:**
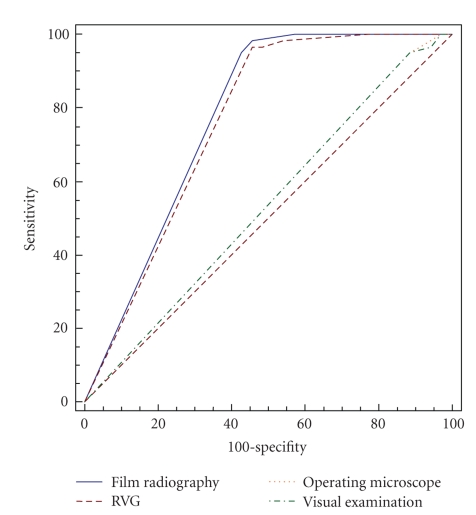
ROC curve for 3rd observer.

Two ROC curves are illustrated. The first ROC curve (Figure 1) is illustrated by considering assessments of 1st observer due to no statistically significant difference between 1st and 2nd observers and the second ROC curve (Figure 2) is illustrated for 3rd observer. Areas under the ROC curve (*A*_*z*_) and standard errors are shown in Table 4 and analysis of *A*_*z*_ values are shown in Table 5.

For both 1st and 3rd observers, no statistically significant difference was found between operating microscope-unaided visual examination and film radiography (Insight)-RVG in 95% confidence interval according to pairwise comparison (*P* < .05). There was a statistically significant difference between operating microscope-film radiography, operating microscope-RVG, unaided visual examination-film radiography, unaided visual examination- RVG in 95% confidence interval according to pairwise comparison (*P* < .05) for both 1st and 3rd observers.

## 4. Discussion

The efficiency of operating microscope was compared with unaided visual examination, film and digital intraoral radiography for proximal caries detection according to ROC analysis in this study.

Recently, many researchers have advocated the use of ROC analysis to assess diagnostic methods for the detection of dental caries [13]. Validity of ROC analysis can be assessed by increasing the number of tooth surfaces, increasing the rating scale, and uniform distribution of caries depths [14]. In this study, the sample was relatively large, 5-point rating scale was used, and the distribution of caries depths was not uniform. Area under the ROC curve (*A*_*z*_ value) gives useful information to measure accuracy of a diagnostic system [15]. The highest *A*_*z*_ values belonged to film radiography and RVG for all observers. The *A*_*z*_ values of unaided visual examination and operating microscope were equal and lower than the radiographic methods.

A diagnostic tool should be reliable and valid. Interobserver reliability is an important factor for this aim [16]. On the other hand, training and experience of observers may affect intra- and interobserver agreements [17]. Syriopoulos et al. [18] emphasized that diagnosis of the radiologists was significantly closer to actual lesion depth than that of general practitioners. Two of the observers were the specialists of oral diagnosis and radiology, the other observer was a specialist of restorative dentistry of at least 10 years of experience in this study. No statistically significant difference was found between the two specialists of oral diagnosis and radiology for all diagnostic systems (*P* < .05), but there was a statistically significant difference between the specialist of restorative dentistry and the specialists of oral diagnosis and radiology (*P* < .05). The *A*_*z*_ values were found to be 0.800, 0.793, and 0.650 for film radiography, RVG, and both unaided visual examination and operating microscope, respectively, according to assessments of 1st observer. The *A*_*z*_ values were found to be 0.773, 0.760, 0.533 for film radiography, RVG, and both unaided visual examination and operating microscope, respectively, according to assessments of 3rd observer in this study. The *A*_*z*_ values of 1st observer were higher than 3rd observer for all diagnostic methods. This condition may be due to the fact that the specialists of oral diagnosis and radiology were more experienced than other specialists about diagnostic and radiographic methods.

Due to difficulty of proximal caries diagnosis with only visual examination, the combination of visual inspection and bitewing radiographic images is accepted as a standard procedure in proximal caries detection [5, 19]. Machiulskiene et al. [20] reported that the clinical examination alone detected about 60% of the total number of proximal cavitated dentin lesions, and bitewing examination detected about 90% of these lesions. But they emphasized that the clinical examination is a more effective method in noncavitated enamel lesions. In this study, the radiographic methods were better than clinical examinations for proximal caries diagnosis in conformity with previous studies [19, 21].

The positioning of operating microscope is the most common difficultness. The operator should be careful and not change the position as far as possible. It was reported that the ideal operator zones are in the 7 to 12 o'clock positions for right-handed operators, and 5 to 12 o'clock for left ones. The clinicians should conform these suggestions to use operating microscope effectively [22]. The researchers studied at 12 o'clock position and not changed the position of operating microscope during the examinations in this study.

Currently, magnifying visual aids such as magnification eyeglasses, stereo microscope [23], and also digital imaging [24] with magnification are used in proximal caries detection in some studies and they reported that these methods are effective. However, Haak et al. reported that prism loupe or surgical microscope does not improve the ability to diagnose proximal caries [25]. In this study, the efficiency of operating microscope was evaluated by comparing with unaided visual examination, film and digital intraoral radiography for proximal caries detection according to ROC analysis. No statistically significant difference was found between operating microscope and unaided visual examination (*P* < .05), and there was a statistically significant difference between operating microscope and both two radiographic systems (*P* < .05).

In conclusion, the efficiency of operating microscope was found statistically equal with unaided visual examination and lower than film and digital intraoral radiography according to ROC analysis. Because the operating microscope is expensive and requires equipment and operator experience, according to the results of this in vitro study it can be said that use of this device would not improve to make an accurate diagnosis of proximal caries lesions. However, the accuracies of diagnostic methods with magnifying visual aids should be investigated and clinical usefulness of these methods in dental practice should be discussed in vitro and in vivo with several studies in which the numbers of samples are larger and rating scales are increased by comparing conventional methods for proximal caries detection.

## Figures and Tables

**Table 1:** Criteria used for evaluations.

Scores	Visual examination & operating microscope	Radiographic	Histological
0	No lesion	Sound	Sound
1	Enamel opacity with smooth surface	Radiolucency in enamel	Caries in enamel
2	Enamel opacity with rough surface	Radiolucency in dentino-enamel junction	Caries in dentino-enamel junction
3	Cavitation restricted to the enamel	Radiolucency in the outer half of the dentine	Caries in the outer half of the dentine
4	Cavitation extending into dentine	Radiolucency in the inner half of the dentine	Caries in the inner half of the dentine

**Table 2:** Histological examination of the teeth.

Scores	No. of tooth surfaces	Percent (%)
Score 0	61	63.54
Score 1	3	3.12
Score 2	12	12.5
Score 3	2	2.09
Score 4	18	18.75

**Table 3:** Results of Scheffe test.

Observers	Groups	Mean difference	Standard error	*P* value	Asymptotic 95% confidence interval	
					Lower bound	Upper bound
1	2	−0.057	0.089	.811	−0.27	0.16
	3	0.531(*)	0.089	.000	0.31	0.75
2	1	0.057	0.089	.811	−0.16	0.27
	3	0.589(*)	0.089	.000	0.37	0.81
3	1	−0.531(*)	0.089	.000	−0.75	−0.31
	2	−0.589(*)	0.089	.000	−0.81	−0.37

* The mean difference is significant at the 0.05 level.

**Table 4:** The *A*_*z*_ values and standard errors for 1st and 3rd observers.

	Test result variable (s)	Area	Std. error (a)	Asymptotic 95% confidence interval	
				Lower bound	Upper bound
1st Observer	Unaided visual examination	0.650	0.060	0.546	0.745
	Operating microscope	0.650	0.060	0.546	0.744
	Film radiography	0.800	0.050	0.706	0.875
	RVG	0.793	0.051	0.698	0.869
3rd Observer	Unaided visual examination	0.533	0.062	0.428	0.635
	Operating microscope	0.533	0.062	0.429	0.636
	Film radiography	0.773	0.052	0.677	0.853
	RVG	0.760	0.054	0.662	0.841

**Table 5:** Pairwise comparisons of *A*_*z*_ values.

	Pairwise	Difference between area	Std. error (a)	*P* value	Asymptotic 95%	
					confidence interval	
					Lower bound	Upper bound
1st Observer	Operating microscope-unaided visual examination	0.000	0.051	.996	−0.099	0.099
	Operating microscope-film radiography	0.150	0.072	.036	0.010	0.291
	Operating microscope-RVG	0.143	0.072	.048	0.001	0.285
	Unaided visual examination-film radiography	0.150	0.072	.038	0.009	0.291
	Unaided visual examination-RVG	0.143	0.073	.050	0.000	0.285
	Insight-RVG	0.007	0.054	.896	−0.099	0.113

3rd Observer	Operating microscope-unaided visual examination	0.001	0.036	.984	−0.070	0.071
	Operating microscope-film radiography	0.240	0.078	.002	0.087	0.393
	Operating microscope-RVG	0.226	0.078	.004	0.074	0.379
	Unaided visual examination-film radiography	0.241	0.078	.002	0.088	0.394
	Unaided visual examination-RVG	0.227	0.078	.003	0.075	0.380
	Film radiography-RVG	0.014	0.047	.772	−0.078	0.106
